# Residual dentin thickness in maxillary first premolars with palatal groove after endodontic and restorative procedures: An e-Vol DX analysis

**DOI:** 10.4317/jced.62794

**Published:** 2025-06-01

**Authors:** Gilberto Siebert Filho, Helder Fernandes de Oliveira, Alline Soares Vaz, Karolina Kellen Matias, Orlando Aguirre Guedes, Jesus Djalma Pécora, Álvaro Henrique Borges, Rafael Ratto de Moraes

**Affiliations:** 1 Department of Endodontics, School of Dentistry, University of Cuiabá, Cuiabá, Brazil; 2 Department of Endodontics, School of Dentistry, Evangelical University of Goiás, Anápolis, Brazil; 3 Department of Oral Biology, School of Dentistry, Pontifical Catholic University of Goiás, Goiânia, Brazil; 4 Department of Restorative Dentistry, School of Dentistry, University of São Paulo, Ribeirão Preto, Brazil; 5 School of Dentistry, Federal University of Pelotas, Pelotas, Brazil

## Abstract

**Background:**

This study aimed to measure residual dentin thickness in maxillary first premolars with a palatal groove after root canal instrumentation, filling material removal, and post space preparation using e-Vol DX, an advanced CBCT imaging analysis software.

**Material and Methods:**

Fourteen extracted maxillary first premolars with a palatal groove on the buccal root were selected. Dentin thickness was measured at 4 stages: initial (M1), after instrumentation (M2), after filling material removal (M3), and after post space preparation (M4). Measurements were taken in 3 root regions: cervical (1 mm coronal to the groove), middle (at the groove’s deepest point), and apical (2 mm apical to the groove). Data were analyzed using ANOVA and Tukey’s post hoc test (α = 5%).

**Results:**

Significant reductions in dentin thickness were found in all walls across treatment phases. In the palatal wall, dentin thickness dropped below 1 mm after instrumentation, retreatment, and post space preparation. In the buccal and mesial walls, thickness fell below 1 mm after retreatment and/or post space preparation. The distal wall was less affected. The average groove depth was 0.66 ± 0.20 mm, and average groove length was 5.72 ± 1.65 mm.

**Conclusions:**

Post space preparation in maxillary first premolars with palatal groove on the buccal root significantly reduces dentin thickness, especially in the palatal wall, increasing the risk of root weakening. Clinicians should carefully assess the indication of intraradicular posts in such cases to avoid potential complications.

** Key words:**Cone-beam computed tomography, e-vol DX software, Maxillary first premolar, residual dentin thickness, root thickness.

## Introduction

The success of endodontic treatment relies on several critical steps, including the thorough chemical and mechanical cleaning of the root canals and their hermetic filling (1,2). Additionally, a comprehensive understanding of root and canal morphology is vital for ensuring effective treatment outcomes. Neglecting these factors is a common cause of treatment failure, often leading to persistent infections or procedural complications (1,3).

The palatal groove on the buccal root of maxillary premolars is a significant anatomical feature (2,4). Two main theories have been proposed to explain the origin of this structure. Gher and Vernino (4) suggest that the groove arises from the developmental tendency of root to bifurcate. In contrast, Ten Cate (5) hypothesizes that the groove forms during tooth germ development through the eccentric expansion of the epithelial diaphragm, wherein two horizontal epithelial flaps develop, proliferate, and merge, leading to groove formation. The prevalence of the palatal groove varies significantly across the studies. Booker and Langhlin (6) reported an occurrence of 100% in their sample, while Tamse *et al*. (7) observed a prevalence of 97%. Gher and Vernino (4) identified an incidence of 78%, and Joseph *et al*. (8) reported a prevalence of 62%.

This groove is characterized by specific dimensions, with a reported depth of 0.43 ± 0.14 mm (9), and length of 4.71 ± 2.08 mm (10). Its point of origin can vary, beginning either before the furcation (0.48 ± 0.43 mm), after the furcation (0.78 ± 0.99 mm), or precisely at the furcation region (10). The palatal wall thickness of the buccal root has been measured at 1.17 ± 1.08 mm in the cervical third, 0.97 ± 0.05 mm in the middle third, and 0.85 ± 0.06 mm in the apical third (11). The presence of the palatal groove, combined with reduced palatal wall thickness, poses significant challenges in endodontic treatment (12). These challenges are particularly pronounced during canal preparation, removal of filling material in retreatment cases, and preparation for intraradicular posts. Excessive wear in this region can lead to complications such as perforations, potentially compromising the success of treatment (2,7,13).

Studies utilizing cone-beam computed tomography (CBCT) have highlighted the intricate complexity of root anatomy in maxillary premolars (9,14). This technology has proven invaluable for diagnosing, identifying etiological factors, and treating teeth with complex anatomical variations, establishing CBCT as an essential tool for successful endodontic treatment and clinical decision-making (9,14,15). Additionally, advancements in CBCT software, such as e-Vol DX (CDT Software, Bauru, SP, Brazil), have significantly enhanced the quality of tomographic images. These innovations include dynamic navigation in multiple planes, brightness and contrast adjustments, thickness and interval adjustments between slices, artifact reduction, and the application of various filters, among other functionalities (16).

Research on root wall thickness after post space preparation highlights the complexity and precision required during these procedures, particularly in teeth with a palatal groove (12). In such cases, one of the walls can become critically thin, with thicknesses falling below 1 mm (12,17). Studies by Katz *et al*. (18) and Ghoddusi *et al*. (19) reveal that while the dentin thickness in the buccal root is generally equal to or greater than that of the palatal root, the palatal wall of the buccal root - where the palatal groove may be present - often exhibits reduced thickness. These findings emphasize the importance of avoiding excessive preparation in this region to prevent structural compromise (20).

The aim of this study was to measure residual dentin thickness in maxillary first premolars with palatal groove after root canal instrumentation, root filling material removal and post-space preparation procedures, using a novel advanced CBCT analysis software (e-Vol DX). The hypothesis was that each subsequent treatment stage would progressively reduce dentin thickness and potentially result in values below the minimum safety threshold in the region affected by the groove.

## Material and Methods

The research protocol was revised and approved by the Local Research Ethics Committee (approval number 2.892.241).

- Sample size calculation

An a priori power analysis was performed using G*Power software v.3.1.9.4 (University of Kiel, Germany) to determine the minimum required sample size. Based on conventional parameters (α = 0.05, power = 0.99), the analysis indicated that a sample size of 14 specimens would be sufficient to ensure adequate statistical power for detecting significant effects.

- Sample selection

This study included 40 human first maxillary premolars selected from a tooth bank. The teeth were initially immersed in 5% sodium hypochlorite (NaOCl; Rioquímica, São José do Rio Preto, SP, Brazil) for 30 minutes to facilitate the removal of organic residues, and then rinsed in running water for 1 hour and dried with absorbent paper.

In the first phase, a visual inspection of the external structures of each tooth was conducted. Teeth that were intact (free from caries, anomalies and restorations), with two fully formed roots and bifurcation located at the junction of the cervical and middle thirds or in the middle third (12), were included in this study. Teeth with prosthetic crowns were excluded. After this initial screening, 20 teeth were excluded, leaving 20 for further analysis. These remaining teeth were subjected to tomographic evaluation.

- CBCT image acquisition

The teeth were mounted in 5 cm-diameter plastic bases filled with silicone (PolyStic, Pulvitec, São Paulo, SP, Brazil), forming 4 blocks of 5 teeth each. CBCT images were acquired using a 13-bit PreXion 3D Elite scanner (PreXion Inc., San Mateo, CA, USA). The scanner parameters were as follows: 0.1 mm slice thickness (dimensions 1.17 mm × 1.57 mm × 1.925 mm), 56 mm field of view (FOV), 0.1 mm voxel, 90 kVp tube voltage, 4 mA tube current, and 33.5-second exposure time. The images were initially viewed using the PreXion 3D Viewer software (TeraRecon Inc., Foster City, CA, USA) on a workstation running the Windows XP Professional SP-2 (Microsoft Corp., Redmond, WA, USA). The workstation was equipped with an Intel Core 2 Duo-6300 1.86 GHz processor (Intel Corp., Santa Clara, CA, USA), an NVIDIA GeForce 6200 Turbo Cache video card (NVIDIA Corporation, Santa Clara, CA, USA), and an EIZO Flexscan S2000 monitor with 1600 × 1200-pixel resolution (EIZO NANAO Corp., Hakusan, Japan). The tomographic images were then transferred to an Asus U474 laptop running Windows 10 (Microsoft Corp., Redmond, WA, USA) with an Intel Core i7 2.4 GHz processor (Intel Corp., Santa Clara, CA, USA) and post-processed using the e-Vol DX software (CDT Software, Bauru, SP, Brazil).

In the second phase, additional inclusion criteria were applied based on tomographic evaluation. Teeth were included if they had one canal per root, a palatal groove on the buccal root, a total length between 19 and 24 mm, a minimum curvature radius of 8 mm on the buccal root (21), no internal or external calcifications or resorptions, groove depth ≥ 0.24 mm, groove length ≥ 3 mm, and a palatal wall thickness of the buccal root ≥ 0.7 mm (9). Endodontically treated teeth, and teeth with intraradicular posts were excluded. After this stage, 14 teeth metall the criteria and were selected as the final study sample.

- Root canal instrumentation

Standard access cavities were prepared using a #1013 round diamond bur (KG Sorensen, Barueri, SP, Brazil), and a #3083 bur (KG Sorensen). Both burs were driven in a high-speed handpiece (Kavo Ind. Com. Ltda., Joinville, SC, Brazil) under refrigeration. The buccal root canal was explored using stainless steel #10 K-Files (Dentsply Maillefer, Ballaigues, Switzerland). The work length (WL) of each tooth was established using CBCT images. The root canals were enlargement using instruments from the ProTaper Gold® system (Dentsply Maillefer), according to the manufacturer’s instructions, at 300 rpm and 2.5 N/cm2 torque. The sequence used was Sx (#19/.04), S1 (#18/.02), S2 (#20/04), F1 (#20/.07), F2 (#25/.08), F3 (#30/.09) and F4 (#40/.06). Root canals were irrigated with 2.5% NaOCl (Rioquímica) at each instrument change during instrumentation. A new set of instruments was used for every five root canals. After instrumentation, the root canals were dried with paper points (Dentsply Maillefer) and irrigated with 17% EDTA (Biodinâmica, Ibiporã, PR, Brazil), which was left in the canal for 3 minutes to remove the smear layer. A final irrigation was performed with 2.5% NaOCl (Rioquímica), after which the canals were dried again with absorbent paper points (Dentsply Maillefer).

- Root filling material removal

The buccal root canal was filled using ProTaper Gold gutta-percha cones (Dentsply Maillefer) and AH Plus endodontic sealer (Dentsply Maillefer) with the hybrid Tagger technique. Condensation was performed with a #60 McSpadden compactor (Dentsply Maillefer), driven using an INTRAMatic 2068 contra-angle and an INTRAMatic 181DBN micromotor (KaVo) at 15,000 RPM. Radiographs were taken in bucco-lingual and mesio-distal directions to confirm the quality of the obturation. Gutta-percha cutting and vertical condensation were completed using Paiva instruments (Golgran, São Caetano do Sul, SP, Brazil). To remove cement and gutta-percha residues, the cavity was cleaned with orange oil (Citrol; Biodinâmica). Root canal filling material removal was carried out 15 days after obturation. For filling material removal, the ProTaper Retreatment D1 (#30/.09), D2 (#25/.08), and D3 (#20/.07) instruments (Dentsply Maillefer) were used. The retreatment files were operated at a constant speed of 500 rpm for D1 and 400 rpm for D2 and D3, with a torque of 3 Ncm. To complete the gutta-percha removal, additional instrumentation was performed using the F5 (#50/.05) instrument of the ProTaper Gold® system (Dentsply Maillefer). The filling material removal was performed based on the previous WL. A new set of instruments was used for every five root canals. During root canal filling material removal, root canals were irrigated with 2.5% NaOCl (Rioquímica) after each instrument change. Final irrigation consisted of 17% EDTA (Biodinâmica) for 3 min, followed by 2.5% NaOCl (Rioquímica). Retreatment was considered complete when no debris of root filling material were observed on the instrument surfaces or in the irrigation solution.

- Post space preparation

After 7 days, the specimens were prepared for the insertion of fiberglass posts. The preparation was performed using White Post DC 2 system burs (FGM, Joinville, SC, Brazil). These burs feature a double-taper design, with a tip diameter of 1.05 mm and a maximum diameter of 1.8 mm. The preparation length extended from the buccal cusp to 3 mm apical to the deepest part of the groove or until resistance prevented further penetration. Throughout the procedure, the canal was irrigated with 2.5% NaOCl. Each bur was used to prepare up to 7 teeth and was activated using an INTRAMatic 2068 contra-angle and an INTRAMatic 181DBN micromotor (KaVo), operating at a rotation speed of 15,000 RPM. Since the objective was to evaluate wall thickness after post space preparation, the fiberglass posts were neither tested nor cemented in the canal.

All endodontic e restorative procedures were performed by the same operator, a specialist in endodontics with 10 years of clinical experience.

- Measurement of residual dentin thickness

Residual dentin thickness in the buccal, mesial, distal, and palatal walls of the buccal root of the maxillary first premolars was measured at four time points: before root canal instrumentation (initial; T1), after instrumentation (T2), after filling material removal (T3), and after post space preparation (T4). Measurements were taken in three regions of the buccal root: 1 mm coronal to the groove’s deepest point (cervical), at the groove’s deepest point (middle) and 2 mm apical to the groove’s deepest point (apical).

For image analysis, each tooth was isolated using the cropping tool and aligned with the axial, coronal and sagittal planes. Groove depth was determined by drawing a line from the outer edge of the palatal wall to the deepest portion of the groove. To minimize parallax error, the buccal canals were aligned along the axiall plane, and the sagittal and coronal planes were used to ensure the long axis of the tooth remained parallel to the ground.

Dentin thickness was measured on CBCT images using the measurement filter of the e-Vol DX software (CDT Software), following the method described by Bueno *et al*. (16). This involved defining precise anatomical points along the edges of the root canal walls and calibrating grayscale intensity to determine the intermediate position. Thin 0.10-mm slices were generated from the 3D reconstructions, and measurements were taken on the axial plane. The correct 3D position was confirmed were using multiplanar reconstruction and a positioning guide. Dimensional calibration ensured consistency between 2D and 3D modes.

The same procedure was repeated on the opposite side of each root, and the dentin thickness was measured in all four directions - buccal, mesial, distal and palatal - at the three specified levels (cervical, middle and apical) across the four stages (T1 - T4).

All imaging analysis was independently performed by two experienced observers - a radiologist and an endodontist, each with over 10 years of experience and previously calibrated. In cases of disagreement, a third expert was consulted to reach a consensus.

- Statistical analysis 

Statistical analysis was performed using R software (Lucent Technologies, New Jersey, USA). The data on dentin wall thickness measurements were initially tested for normality and homoscedasticity to ensure the assumptions for parametric testing were met. Analysis of Variance (ANOVA) was then conducted, followed by Tukey’s post hoc test, with a significance level set at 5%.

## Results

 Table 1,  Table 2 and  Table 3 summarize the results. Table 1 shows the mean root dentin thickness of the buccal root canal at 1 mm coronal to the groove’s deepest point. A significant reduction in dentin thickness was observed in the buccal wall between the initial (T1) and root filling material removal (T3) and post space preparation phase (*p* < 0.05). In the mesial and distal walls, significant reductions were observed between the T1 and all subsequent phases (*p* < 0.05). In the mesial wall, a significant difference was also noted between the instrumentation (T2) and T3 phases. In the palatal wall, significant reductions were found between the T1 and all subsequent phases (*p* < 0.05). No significant difference was observed between T2 and T3 (*p* > 0.05), but both differed significantly from the T3 phase, where the remaining dentin thickness dropped below 1 mm (*p* < 0.05).

 Table 2 shows the mean thickness at the groove’s deepest point. In the buccal wall, significant reductions were observed between the T1 and T4 phases (*p* < 0.05), with no significant difference between T2 and T3 (*p* > 0.05). In the distal wall, significant differences were found between the T1 and all subsequent phases (*p* < 0.05). In the palatal wall, dentin thickness dropped below 1 mm across all root canal preparation phases.

 Table 3 shows the mean thickness at 2 mm apical to the groove’s deepest point. All walls exhibited significant differences between the T1 and subsequent phases (*p* < 0.05). No significant differences were found between T1 and T2 in any wall (*p* > 0.05). Remaining dentin thickness was reduced to less than 1 mm in the following walls: palatal wall after T3 and T4; buccal wall after T3 and T4; and (3) mesial wall after T4.

The results indicate that the average depth of the palatal groove on the buccal root, measured at its deepest point, was 0.66 ± 0.20 mm, while the average groove length was 5.72 ± 1.65 mm.

**Figure 1:**
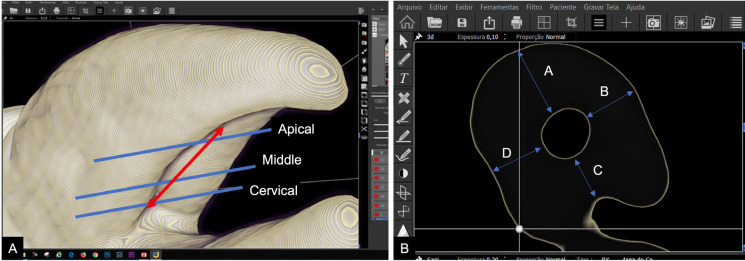
(A) 3D reconstructions showing the measurements sites: 1 mm coronal to the groove (cervical), at the groove’s deepest point(middle), and 2 mm apical to the groove (apical). (B) CBCT image demonstrating the dentin thickness measurement areas on the buccal root using the e-volDX software filter. The measured root walls are identified as follows: A- buccal, B - Mesial, C - Palatal and D - Distal.

Figure 1 presents CBCT scans and 3D reconstructions that illustrate the methodology and the specific e-Vol DX software filter used for measurements in this study.

## Discussion

The thickness of the remaining dentin in the buccal root of maxillary first premolars with a palatal groove was found to be less than 1 mm in all studied regions following post space preparation. Similarly, dentin thicknesses below 1 mm were observed after root filling material removal in the deepest region of the groove and 2 mm apical to it. These findings support that tested hypothesis, confirming that the remaining dentin thickness progressively decreases with each treatment phase, often reaching critical levels.

Studies of anatomical structures should utilize innovative technologies to replicate clinical scenarios more effectively (16,22). Historically, dental anatomy was analyzed using photographs and tooth sections (4,12), where observations of external tooth characteristics made significant contributions (23). To study internal root canal anatomy, root canal casting techniques were developed (24). Radiographs were also extensively used to determine the number and shape of canals; however, their limitation was the inability to provide three-dimensional visualization (25).

Other studies have explored known or previously unexamined scenarios by introducing new technologies for anatomical assessments. Techniques such as tooth clearing (3), stereoscopic loupes (13), clinical microscopes (7,12), scanning electron microscopy (26), cone-beam computed tomography (CBCT) (27), and micro-computed tomography (10) have significantly advanced the field of anatomical investigation. High-tech equipment has become increasingly relevant, particularly when precise investigations are required or when known phenomena must be analyzed in greater detail, especially in *in vivo* studies. Recent advancements in software development have further enhanced the precision of established technologies, enabling new possibilities for diagnosis, treatment planning, and the detailed visualization of specific anatomical structures across different tooth groups (16).

The e-Vol DX software leverages CBCT imaging to conduct precise analyses. CBCT offers the unique advantage of allowing the reuse of teeth after various types of preparation, enabling quick and safe comparisons across various treatment modalities (9,10). Additionally, CBCT can be utilized for both extracted teeth in research and *in vivo* investigations, a capability not possible with micro-CT (16,22). Another benefit of CBCT is its accessibility, as it is widely employed in clinical treatments, particularly in endodontics (22). Given the numerous applications of this technology, the present study evaluated the relationship between endodontic and restorative treatment stages and the palatal groove, an anatomical structure present on the buccal root of maxillary first premolars. Unlike the study by *Pi*lo *et al*. (12), which broadly assessed wall thickness during endodontic and post space preparation, this research specifically focused on the palatal groove region. The analysis targeted dentin wall thickness in the groove region using anatomical references, such as the cementoenamel junction or furcation, rather than random areas. Furthermore, the methodology employed CBCT and advanced software (e-Vol DX), offering a more sophisticated approach compared to Pilo *et al*. (12), who relied on dentin sections and clinical microscope evaluations to measure wall thickness.

Given that this study also evaluated the initial characteristics of teeth prior to preparation, it provides a basis for comparing its findings on the specific anatomy of the palatal groove with other studies. Lammertyn *et al*. (11), using root sections, photomicrography, and enlarged image projection, investigated the anatomical characteristics of the palatal groove on the buccal root of maxillary premolars. Their findings reported an average groove depth of 0.44 mm at the coronal level, 0.34 mm at the middle level, and 0.17 mm at the apical level. In comparison, the current study found a depth groove of 0.66 mm in the deepest groove region, indicating a considerable difference. Lammertyn *et al*. (11) also measured root wall thickness in three distinct regions: 1.41 ± 0.17 mm in the cervical region, 1.13 ± 0.13 mm in the middle region, and 0.77 ± 0.16 mm in the apical region. In this study, the palatal wall thickness in the deepest groove region was 1.26 ± 0.13 mm, more cervical to the groove it was 1.40 ± 0.15 mm, and more apical it was 1.29 ± 0.14 mm. These findings demonstrate that the groove’s central region initially exhibits thinnest dentin. Al-Shahrani *et al*. (10), using micro-CT, evaluated the anatomy of maxillary first premolars and found thinner walls in the palatal groove region, with an average of 0.78 ± 0.14 mm, compared to 1.26 ± 0.13 in the present study. Al-Shahrani *et al*. (10) also reported an average length of 4.71 ± 2.08 mm, whereas this study found a longer groove length of 5.72 ± 1.65 mm. Conversely, Li *et al*. (9) reported palatal wall thicknesses of 0.73 ± 0.15 mm in the coronal third, 0.66 ± 0.12 mm in the middle third, and 0.25 ± 0.06 mm in the apical third of the palatal surface of the buccal root of maxillary premolars. The average groove length in their study was 3.94 ± 0.64 mm. Both the Al-Shahrani *et al*. (10) and Li *et al*. (9) did not specifically evaluate the deepest groove region. Instead, they divided the root into thirds or used other anatomical references, making it unclear whether the values presented by these studies represent the lowest thickness values for this specific structure. However, regarding groove length, the assessment methodologies were similar, allowing for more direct comparisons. Another key distinction that both studies focused exclusively on anatomical evaluations and did not relate their findings to root canal preparations, as was done in the present study. This integration of anatomical analysis with clinical relevance represents a significant contribution of the current investigation.

Katz *et al*. (18) evaluated wall thickness in two-rooted premolars before and after endodontic and post space preparation using a camera adapted to a microscope. Their study found the smallest thickness values in the palatal wall of the buccal root, with measurements ranging from 0.99 mm before preparation to 0.68 mm after post space preparation at the coronal level of the root. At the middle level, the thickness values were 0.78 mm before preparation and 0.66 mm after post space preparation. In the present study, the cervical region thickness before preparation was 1.40 ± 0.15 mm, decreasing to 0.77 ± 0.22 mm after post space preparation. In the deepest groove region, the initial thickness was 1.26 ± 0.13 mm, which reduced to 0.64 ± 0.16 mm following preparation. These findings indicate significant dentin wear in the palatal wall across all three regions evaluated during post space preparation, resulting in dentin thicknesses of less than 1 mm. Additionally, dentin thicknesses below 1 mm were observed after retreatment in the deepest groove region and in the most apical region.

Considering the mechanical aspects related to lateral and occlusal forces, as well as the forces exerted on dental restoration, it is critical to evaluate cases where the remaining dentin thickness is less than 1 mm. Pilo *et al*. (12) emphasized that a dentin thickness below 1 mm after post space preparation compromises root integrity. Similarly, Naumann *et al*. (28), in a meta-analysis on the ferrule effect in endodontically treated teeth prepared for post space, concluded that the ferrule effect and preservation of dental walls are crucial for restoration success and tooth longevity. Naumann *et al*. (29) further demonstrated that teeth with wall thicknesses between 0.5 mm and 0.75 mm, and without a ferrule, exhibited a significantly higher failure rate compared to teeth without a ferrule but with wall thicknesses between 2.4 mm and 2.9 mm. These findings underline the importance of preserving as much dentin as possible to enhance the predictability of rehabilitative treatments in endodontically treated teeth. The ferrule effect, in conjunction with maintaining a dentin greater than 1 mm, is crucial for treatment longevity (12,30) and aligns with the principles of minimally invasive dentistry. Furthermore, when sufficient coronal structure remains, the ferrule effect becomes more significant than the need for an intraradicular post. This may reduce the need for posts and mitigate the risks associated with the reduced root canals thickness observed in this study.

## Conclusions

Endodontic treatment in maxillary first premolars with a palatal groove on the buccal root can be considered safe in terms of dentin thickness after instrumentation. However, caution is necessary during root filing material removal, as dentin thicknesses may fall below 1 mm in some cases. Post space preparation poses the greatest challenge, as it compounds the wear from previous procedures, significantly increasing the risk of reaching critical thicknesses. These findings underscore the importance of carefully evaluating the use of intraradicular post in the buccal canal of maxillary premolars with a palatal groove. When possible, their use should be avoided to reduce the risk of complications and maintain tooth integrity.

## Figures and Tables

**Table 1:** Mean and standard deviation of root dentin thickness (mm) in the different procedures at 1 mm coronal to the groove’s deepest point.

Procedures	Root canal walls			
	Buccal	Mesial	Distal	Palatal
Initial	1.66 ± 0.20^a^	1.72 ± 0.20^a^	1.67 ± 0.18^a^	1.40 ± 0.15^a^
Root instrumentation	1.43 ± 0.20^ab^	1.44 ± 0.14^b^	1.44 ± 0.15^b^	1.15 ± 0.26^b^
Filling material removal	1.26 ± 0.25^bc^	1.30 ± 0.15^bc^	1.38 ± 0.15^b^	1.01 ± 0.24^b^
Post space preparation	1.18 ± 0.25^c^	1.15 ± 0.18^c^	1.28 ± 0.23^b^	0.77 ± 0.22^c^

In columns, different letters indicate significant differences between the root canal preparation phases (*p* <0.05).

**Table 2:** Mean and standard deviation of root dentin thickness (mm) in the different procedures at the groove’s deepest point.

Procedures	Root canal walls			
	Buccal	Mesial	Distal	Palatal
Initial	1.51 ± 0.19^a^	1.61 ± 0.19^a^	1.62 ± 0.19^a^	1.26 ± 0.13^a^
Root instrumentation	1.29 ± 0.19^ab^	1.32 ± 0.14^b^	1.38 ± 0.15^b^	0.99 ± 0.16^b^
Filling material removal	1.11 ± 0.26^bc^	1.22 ± 0.20^bc^	1.31 ± 0.20^b^	0.86 ± 0.16^b^
Post space preparation	1.00 ± 0.28^c^	1.04 ± 0.26^c^	1.22 ± 0.23^b^	0.64 ± 0.16^c^

In columns, different letters indicate significant differences between the root canal preparation phases (*p* <0.05).

**Table 3:** Mean and standard deviation of root dentin thickness (mm) in the different procedures at 2 mm below the groove’s deepest point.

Procedures	Root canal walls			
	Buccal	Mesial	Distal	Palatal
Initial	1.35 ± 0.17^a^	1.40 ± 0.15^a^	1.57 ± 0.20^a^	1.29 ± 0.14^a^
Roo instrumentation	1.14 ± 0.20^b^	1.15 ± 0.26^b^	1.34 ± 0.25^b^	1.01 ± 0.13^b^
Filling material removal	0.94 ± 0.27^bc^	1.01 ± 0.24^b^	1.18 ± 0.21^b^	0.90 ± 0.17^bc^
Post space preparation	0.86 ± 0.19^c^	0.77 ± 0.22^c^	1.17 ± 0.25^b^	0.84 ± 0.20^c^

In columns, different letters indicate significant differences between the root canal preparation phases (*p* <0.05).

## Data Availability

The datasets used and/or analyzed during the current study are available from the corresponding author.
